# Supplementary material for: Characterization of the Technofunctional Properties and Three-Dimensional Structure Prediction of 11S Globulins from Amaranth (Amaranthus hypochondriacus L.) Seeds
Source: Foods. 2023 Jan 19;12(3):461. doi: 10.3390/foods12030461 (PMC9914310; doi:10.3390/foods12030461)
Supplement: Supplementary file 1 [file foods-12-00461-s001.zip › foods-2112620-supplementary.pdf]

foods

MDPI

Article

Characterization of the Technofunctional Properties and Three-Dimensional Structure Prediction of 11S Globulins from Amaranth (*Amaranthus hypochondriacus* L.) Seeds

Jorge Aguilar-Padilla [1,2,3], Sara Centeno-Leija [3], Esaú Bojórquez-Velázquez [4], José M. Elizalde-Contreras [4], Eliel Ruiz-May [4], Hugo Serrano-Posada [3,*] and Juan Alberto Osuna-Castro [2,*]

[1] Facultad de Ciencias Químicas, Universidad de Colima, Carr. Colima-Coquimatlán km. 9, Coquimatlán 28400, Colima, México; jaguilar17@ucol.mx
[2] Facultad de Ciencias Biológicas y Agropecuarias, Universidad de Colima, Carr. Colima-Manzanillo km. 40, Tecomán 28100, Colima, México
[3] Consejo Nacional de Ciencia y Tecnología, Laboratorio de Biología Sintética, Estructural y Molecular, Laboratorio de Agrobiotecnología, Tecnoparque CLQ, Universidad de Colima, Carretera Los Limones-Loma de Juárez, Colima 28629, Colima, México; scenteno0@ucol.mx
[4] Red de Estudios Moleculares Avanzados, Instituto de Ecología A.C., Cluster BioMimic®, Carretera Antigua a Coatepec 351, El Haya, Xalapa, Veracruz 91073, México; esau.bojorquez@inecol.mx (E.B.-V.); jose.elizalde@inecol.mx (J.M.E.-C.); eliel.ruiz@inecol.mx (E.R.-M.)
* Correspondence: hserrano0@ucol.mx (H.S.-P.); osuna_juan@hotmail.com (J.A.O.-C.)

Supplementary material

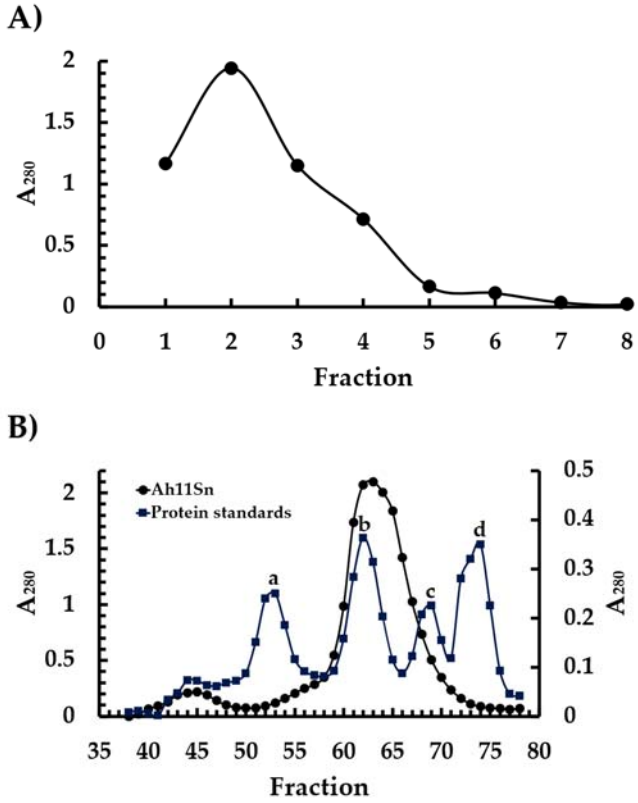

Figure S1. Ah11Sn two-step purification. A) Ionic exchange chromatography using DEAE-Sepharose resin. B) Molecular exclusion chromatography using Sephacryl-S300 resin with gel filtration protein standards (in kDa) (a: Thyroglobulin 670, b: γ-globulin 158, c: ovalbumin 44, d: myoglobin 17).

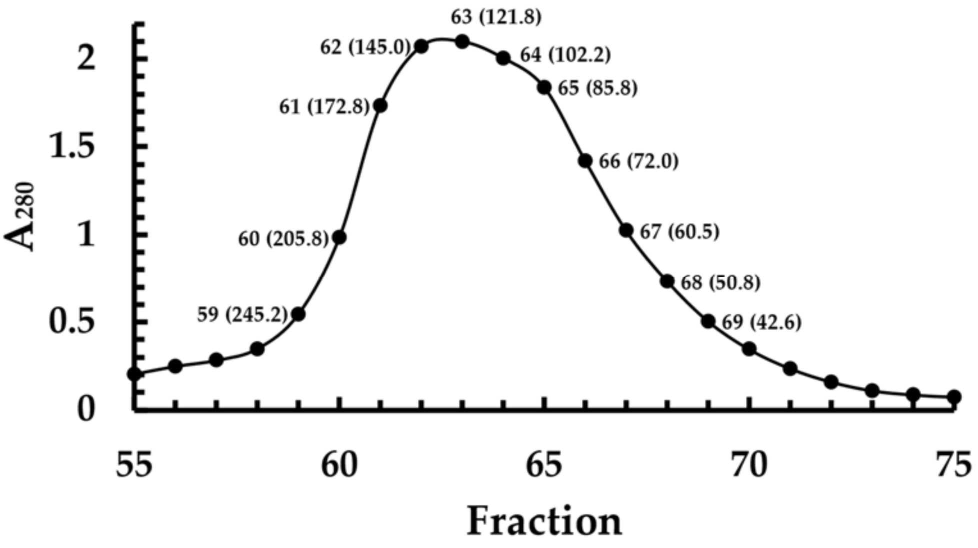

Figure S2. Elution profile of Ah11Sn applied to Sephacryl-S300. Labels indicate the fraction numbers and the corresponding relative molecular weight (kDa) in parentheses.

Table S1. Protein identification of Ah11Sn gel bands analyzed by LC-MS/MS.

Band[1]	Protein	Accession No.[2]	Orthologue ID[3]	Mr(kDa)[4] Exp.	Mr(kDa)[5] Theor.	PM/SC(%)/PSMs[6]	Identified peptides
**7S1**	Vicilin	AHYPO_018839-RA	VCL6_JUGRE	34.6	60.9	1/3/4	GHVMVIPAGVTAYLVNR
**7S2**	Vicilin	AHYPO_018839-RA	VCL6_JUGRE	33.6	60.9	5/10/207	GHVMVIPAGVTAYLVNR
							GHVMVIPAGVTAYLVNRGNNEK
							LVIVKLLNPVSNPSGK
							QSNAFGTLFETDFDDR
							QSNAFGTLFETDFDDRR
	11S globulin	AHYPO_021282-RA AH007202-RA	CONA2_LUPAN		77.6	4/12/38	ALLGVTNPGCPETFEYGSSEPFSSER
							QLLSQAFGIEPETVSK
							TQVEPNGLFLPHYNNAPSISYVIR
							VEGDLGLLIPEWDREESR
	11S globulin	AH017742-RA	11S2_SESIN		45.7	3/13/45	AMPVQVLINSYQISPR
							VSEGEMFVIPQYFAVSAR
							VTVQPNSLTLPNFFPFPR
**11Sα1**	11S globulin	AH017742-RA	11S2_SESIN	29.6	45.7	5/22/111	IECEGGLIELWDENEDQYVCSGVHAMR
							VTVQPNSLTLPNFFPFPR
							AMPVQVLINSYQISPR
							LTSSEPSDRIECEGGLI-
							ELWDENEDQYVCSGVHAMR
							VSEGEMFVIPQYFAVSAR

	11S globulin	AHYPO_021282-RA AH007202-RA	CONA2_LUPAN		77.6	7/18/56	ALLGVTNPGCPETFEYGSSEPFSSER
							QLLSQAFGIEPETVSK
							GLPEQVVMNSYGLSR
							LTSLNSQKLPILNYLQLSAEK
							TQVEPNGLFLPHYNNAPSISYVIR
							GKALLGVTNPGCPETFEYGSSEPFSSER
							VEGDLGLLIPEWDREESR
	Agglutinin	AHYPO_007409-RA	Q38719_AMAHP		30.1	3/16/42	YQSDNIQQYGLLQFSADK
							LSTDNWILVDGNDPR
							SDVHDFNVISLLNMQK
	11S globulin	AHYPO_001411-RA	JUGN4_JUGNI		55.4	4/14/30	IQIVNDQGQSVFDEELSR
							SLPIDVVSNIYQISR
							LLAESFGVSEEIAQK
							IQAEAGLTEVWDSNEQEFR
**11Sα2**	11S globulin	AHYPO_021282-RA AH007202-RA	CONA2_LUPAN	27.6	77.6	22/44/455	LTIVTLFDTLNNQNQLDDILR
							ALLGVTNPGCPETFEYGSSEPFSSER
							GKALLGVTNPGCPETFEYGSSEPFSSER
							ALLGVTNPGCPETFEYGSSEPFSSERDLR
							TQVEPNGLFLPHYNNAPSISYVIR
							LTSLNSQKLPILNYLQLSAEK
							LTRDTQCRIDCQIDQLSANEPNIR
							IDCQIDQLSANEPNIR
							QLLSQAFGIEPETVSKIQGQNDDR
							QLLSQAFGIEPETVSK
							LVFDDMVQEGQLLVVPQNFVVLK
							QLLSQAFGIEPETVSKIQGQNDDRGAIIR
							VEGDLGLLIPEWDREESR
							IFSENNILSGFDRQLLSQAFGIEPETVSK
							GKALLGVTNPGCPETFEYGSSEPFSSER-DLR
							RVYQGHIVALPAGVSK
							GLPEQVVMNSYGLSR
							VYQGHIVALPAGVSKWFYNDGQDR
							VYQGHIVALPAGVSK
							AGQEGLEWVAFLTSDEAMISPLAGR
							IQAEAGVNEIWDPREQKEFQCAGVTVVR

							ALLGVTNPGCPETFEYGSSEPFSSER-DLRRPGHK
	11S globulin	AH017742-RA	11S2_SESIN		45.7	5/2261	VTVQPNSLTLPNFFPFPR
							IECEGGLIELWDENEDQYVCSGVHAMR
							AMPVQVLINSYQISPR
							VSEGEMFVIPQYFAVSAR
							LTSSEPSDRIECEGGLI-ELWDENEDQYVCSGVHAMR
	11S globulin	AHYPO_001411-RA	JUGN4_JUGNI		55.4	4/14/36	IQAEAGLTEVWDSNEQEFR
							IQIVNDQGQSVFDEELSR
							SLPIDVVSNIYQISR
							LLAESFGVSEEIAQK
	11S globulin	AH017744-RA	JUGR4_JUGRE		32.7	4/23/27	IQAEAGLTEVWDSQEQELR
							GISGMLIPGCPESYESGSQQFELGR
							TLAESFGVSEEIAQK
							TLAESFGVSEEIAQKLQGQQDER
**11Sβ1**	11S globulin	AHYPO_021282-RA AH007202-RA	CONA2_LUPAN	23.6	77.6	10/24/92	ALLGVTNPGCPETFEYGSSEPFSSER
							VYQGHIVALPAGVSK
							IDCQIDQLSANEPNIR
							GLPEQVVMNSYGLSR
							QLLSQAFGIEPETVSK
							ITENIDDPEKADVFNPQGGR
							TQVEPNGLFLPHYNNAPSISYVIR
							VEGDLGLLIPEWDREESR
							RVYQGHIVALPAGVSK
							YGRQELTVFSPSEEFQR
	11S globulin	AHYPO_001411-RA	JUGN4_JUGNI		55.4	5/19/74	IQAEAGLTEVWDSNEQEFR
							FYLAGKPQQEHSGEHQFSR
							IQIVNDQGQSVFDEELSR
							LAVNVDDPSKADVYTPEAGR
							SLPIDVVSNIYQISR
	11S globulin	AH017742-RA	11S2_SESIN		45.7	2/8/50	AMPVQVLINSYQISPR
							TTSSPMKSPLVGYTSVFR
	11S globulin	AH017743-RA	JUGR4_JUGRE		13.2	3/30/36	IQIVNDQGQSVFDDELTK
							SLPVDVVSNMYQISR
							GRIQIVNDQGQSVFDDELTK
**11Sβ2**	11S globulin	AH017742-RA	11S2_SESIN	21.8	45.7	5/19/428	AMPVQVLINSYQISPR
							VTVQPNSLTLPNFFPFPR

							TTSSPMKSPLVGYTSVFR
							VSEGEMFVIPQYFAVSAR
							AMPVQVLINSYQISPRDAQELK
	11S globulin	AH017743-RA	JUGR4_JUGRE		13.2	4/42/82	GRIQIVNDQGQSVFDDELTK
							IQIVNDQGQSVFDDELTK
							IQIVNDQGQSVFDDELTKGQLVVVPQN-
							FAIIK
							SLPVDVVSNMYQISR
	11S globulin	AHYPO_001411-RA	JUGN4_JUGNI		55.4	6/22/69	IQAEAGLTEVWDSNEQEFR
							IQIVNDQGQSVFDEELSR
							SLPIDVVSNIYQISR
							LLAESFGVSEEIAQK
							FYLAGKPQQEHSGEHQFSR
							LAVNVDDPSKADVYTPEAGR
	11S globulin	AHYPO_021282-RA AH007202-RA	CONA2_LUPAN		77.6	10/27/55	ALLGVTNPGCPETFEYGSSEPFSSER
							IDCQIDQLSANEPNIR
							QLLSQAFGIEPETVSK
							GLPEQVVMNSYGLSR
							YGRQELTVFSPSEEFQR
							LTSLNSQKLPILNYLQLSAEK
							RVYQGHIVALPAGVSK
							VEGDLGLLIPEWDREESR
							TQVEPNGLFLPHYNNAPSISYVIR
							ITENIDDPEKADVFNPQGGR
	11S globulin	AH017744-RA	JUGR4_JUGRE		32.7	3/20/32	IQAEAGLTEVWDSQEQELR
							TLAESFGVSEEIAQK
							GISGMLIPGCPESYESGSQQFELGR
**11Sβ3**	11S globulin	AH017742-RA	11S2_SESIN	20.4	45.7	7/26/601	IECEGGLIELWDENEDQYVCSGVHAMR
							LTSSEPSDRIECEGGLI-
							ELWDENEDQYVCSGVHAMR
							VTVQPNSLTLPNFFPPFPR
							AMPVQVLINSYQISPR
							TTSSPMKSPLVGYTSVFR
							VSEGEMFVIPQYFAVSAR
							SPLVGYTSVFRAMPVQVLINSYQISPR
	11S globulin	AH017743-RA	JUGR4_JUGRE		13.2	7/61/433	IQIVNDQGQSVFDDELTKGQLVVVPQN-
							FAIIK
							IQIVNDQGQSVFDDELTK
							GRIQIVNDQGQSVFDDELTK

11S globulin	AH017744-RA	JUGR4_JUGRE	32.7	6/44/256	GRIQIVND-<br>QGQSVFDDELTKGQLVVVPQNFAIIK<br>SLPVDVVSNMYQISR<br>TSAIRSLPVDVVSNMYQISR<br>EQAFGLKFNRPETTLFR<br>IQAEAGLTEVWDSQEQELR<br>GISGMLIPGCPESYESGSQQFELGR<br>RFYLAGKPQQEHTEQSR<br>FYLAGKPQQEHTEQSR<br>HEFQQGNECQIDRLTALEPTNRIQAEA-<br>GLTEVWDSQEQELR<br>EGDVHAFPSGVAH-<br>WVYNNGDEPLVLVVFIDNANHANQLD-<br>NNFPK
11S globulin	AHYPO_021282-RA AH007202-RA	CONA2_LUPAN	77.6	13/33/234	LTIVTLFDTLNNQNQLDDILR
11S globulin	AHYPO_001411-RA	JUGN4_JUGNI	55.4	8/22/186	ALLGVTNPGCPETFEYGSSEPFSSER<br>AGQEGLEWVAFLTSDEAMISPLAGR<br>LVFDDMVQEGQLLVVPQNFVVLKK<br>LVFDDMVQEGQLLVVPQNFVVLK<br>LTSLNSQKLPILNYLQLSAEK<br>YGRQELTVFSPSEEFQR<br>KAGQEGLEWVAFLTSDEAMISPLAGR<br>GLPEQVVMNSYGLSR<br>QLLSQAFGIEPETVSK<br>VEGDLGLLIPEWDREESR<br>ITENIDDPEKADVFNPQGGR<br>TQVEPNGLFLPHYNNAPSISYVIR<br>IQIVNDQGQSVFDEELSR<br>GRIQIVNDQGQSVFDEELSR<br>LAVNVDDPSKADVYTPEAGR<br>IQIVNDQGQSVFDEELSRGQLVVVPQN-<br>FAIVK<br>LLAESFGVSEEIAQK<br>SLPIDVVSNIYQISREEAFGLK<br>SLPIDVVSNIYQISR<br>IQAEAGLTEVWDSNEQEFR
Vicilin	AHYPO_010140-RA	VCL21_ARATH	67.2	3/9/145	IIAIFDNPEEAQLQGPLSGPYTSINDLVK<br>TPIAATEFGEILSVDIDDGTGKGK

							TPIAATEFGEILSVDIDDGTGK
**11Sβ4**	11S globulin	AHYPO_021282-RA AH007202-RA	CONA2_LUPAN	19.4	77.6	18/36/588	AGQEGLEWVAFLTSDEAMISPLAGR
							ALLGVTNPGCPETFEYGSSEPFSSER
							GLPEQVVMNSYGLSR
							GLPEQVVMNSYGLSREEAK
							ITENIDDPEKADVFNPQGGR
							KAGQEGLEWVAFLTSDEAMISPLAGR
							LKYGRQELTVFSPSEEFQR
							LPILNYLQLSAEKVNLYQNAIMAPNWK
							LTIVTLFDTLNNQNQLDDILR
							LTSLNSQKLPILNYLQLSAEK
							LTSLNSQKLPILNYLQLSAEKVN-LYQNAIMAPNWK
							LVFDDMVQEGQLLVVPQNFVVLK
							LVFDDMVQEGQLLVVPQNFVVLKK
							QELTVFSPSEEFQRK
							QLLSQAFGIEPETVSK
							TQVEPNGLFLPHYNNAPSISYVIR
							VEGDLGLLIPEWDREESR
							YGRQELTVFSPSEEFQR
	11S globulin	AH017742-RA	11S2_SESIN		45.7	6/26/263	AMPVQVLINSYQISPR
							IECEGGLIELWDENEDQYVCSGVHAMR
							LTSSEPSDRIECEGGLI-ELWDENEDQYVCSGVHAMR
							TTSSPMKSPLVGYTSVFR
							VSEGEMFVIPQYFAVSAR
							VTVQPNSLTLPNFFPFPR
	11S globulin	AHYPO_001411-RA	JUGN4_JUGNI		55.4	7/20/143	GRIQIVNDQGQSVFDEELSR
							IQIVNDQGQSVFDEELSR
							IQAEAGLTEVWDSNEQEFR
							LAVNVDDPSKADVYTPEAGR
							SLPIDVVSNIYQISR
							LLAESFGVSEEIAQK
							SLPIDVVSNIYQISREEAFGLK
	11S globulin	AH017744-RA	JUGR4_JUGRE		32.7	5/39/125	IQAEAGLTEVWDSQEQELR
							EGDVHAFPSGVAH-WVYNNGDEPLVLVVFIDNANHANQLD-NNFPK
							GISGMLIPGCPESYESGSQQFELGR

	11S globulin	AH017743-RA	JUGR4_JUGRE		13.2	4/42/66	TLAESFGVSEEIAQK<br>LTALEPTNRIQAEAGLTEVWDSQEQELR<br>IQIVNDQGQSVFDDELTKGQLVVVPQN-<br>FAIIK<br>GRIQIVNDQGQSVFDDELTK<br>IQIVNDQGQSVFDDELTK
	Vicilin	AHYPO_006304-RA	VCL22_ARATH		61.9	4/12/41	SLPVDVVSNMYQISR<br>MVERPLHIGFITMEPK<br>TMQGPEFATAFGLTEDR<br>IPGGSTFYLVNTGETQR
	Vicilin	AHYPO_010140-RA	VCL21_ARATH		67.2	3/9/29	AGPFEFFGFTTSAHK<br>IIAIFDNPEEAQLQGPLSGPYTSINDLVK<br>TPIAATEFGEILSVDIDDGTGK<br>TPIAATEFGEILSVDIDDGTGKGK
**11Sβ5**	11S globulin	AHYPO_021282-RA AH007202-RA	CONA2_LUPAN	18.3	77.6	15/35/284	AGQEGLEWVAFLTSDEAMISPLAGR<br>ALLGVTNPGCPETFEYGSSEPFSSER<br>GLPEQVVMNSYGLSR<br>GLPEQVVMNSYGLSREEAK<br>ITENIDDPEKADVFNPQGGR<br>KAGQEGLEWVAFLTSDEAMISPLAGR<br>LPILNYLQLSAEKVNLYQNAIMAPNWK<br>LTSLNSQKLPILNYLQLSAEK<br>LVFDDMVQEGQLLVVPQNFVVLK<br>QELTVFSPSEEFQRK<br>QLLSQAFGIEPETVSK<br>RVYQGHIVALPAGVSK<br>TQVEPNGLFLPHYNNAPSISYVIR<br>VEGDLGLLIPEWDREESR<br>YGRQELTVFSPSEEFQR
	11S globulin	AH017742-RA	11S2_SESIN		45.7	4/17/209	VTVQPNSLTLPNFFPFPR<br>AMPVQVLINSYQISPR<br>TTSSPMKSPLVGYTSVFR<br>VSEGEMFVIPQYFAVSAR
	11S globulin	AH017744-RA	JUGR4_JUGRE		32.7	2/15/59	IQAEAGLTEVWDSQEQELR<br>GISGMLIPGCPESYESGSQQFELGR
	11S globulin	AHYPO_001411-RA	JUGN4_JUGNI		55.4	4/11/55	IQIVNDQGQSVFDEELSR<br>GRIQIVNDQGQSVFDEELSR

					SLPIDVVSNIYQISR
					LAVNVDDPSKADVYTPEAGR
11S globulin	AH017743-RA	JUGR4_JUGRE	13.2	3/30/39	IQIVNDQGQSVFDDELTK
					SLPVDVVSNMYQISR
					GRIQIVNDQGQSVFDDELTK
Vicilin	AHYPO_006304-RA	VCL22_ARATH	61.9	4/12/26	MVERPLHIGFITMEPK
					AGPFEFFGFTTSAHK
					TMQGPEFATAFGLTEDR
					IPGGSTFYLVNTGETQR
Vicilin	AHYPO_018839-RA	VCL6_JUGRE	60.9	4/12/19	GHVMVIPAGVTAYLVNR
					EAQELAFASSAEEIER
					VFETQEEEFFFPGPR
					QSNAFGTLFETDFDDRR

[1]Band numbers according to Figure 2. [2]Accession number according to the databases reported previously [17,18]. [3]Orthologue according to the best BLAST match in Swiss-Prot database. [4]Experimental molecular weight (kDa). [5]Theoretical molecular weight (kDa). [6]Peptides Matched/Sequence Coverage/Peptide Spectrum Matches.

Table S2. Validation parameters of amaranth 11S globulins 3D models.

Acc. No.[1]	Procheck	Errat	Verify 3D	EM[2] (kJ/mol)
AHYPO_001411-RA	91.4 % core<br>8.6 % allowed<br>0.0 % generous<br>0.0 % disallowed	97.93 %	82.55 %	-733561.4
AH017742-RA	89.8 % core<br>10.2 % allowed<br>0.0 % generous<br>0.0 % disallowed	90.42 %	71.26 %	-605360.3
AHYPO_021282-RA	88.8 % core<br>11.1 % allowed<br>0.1 % generous<br>0.0 % disallowed	87.345 %	64.31 %	-964234.1

[1]Protein accession number. [2]Energy minimization step.

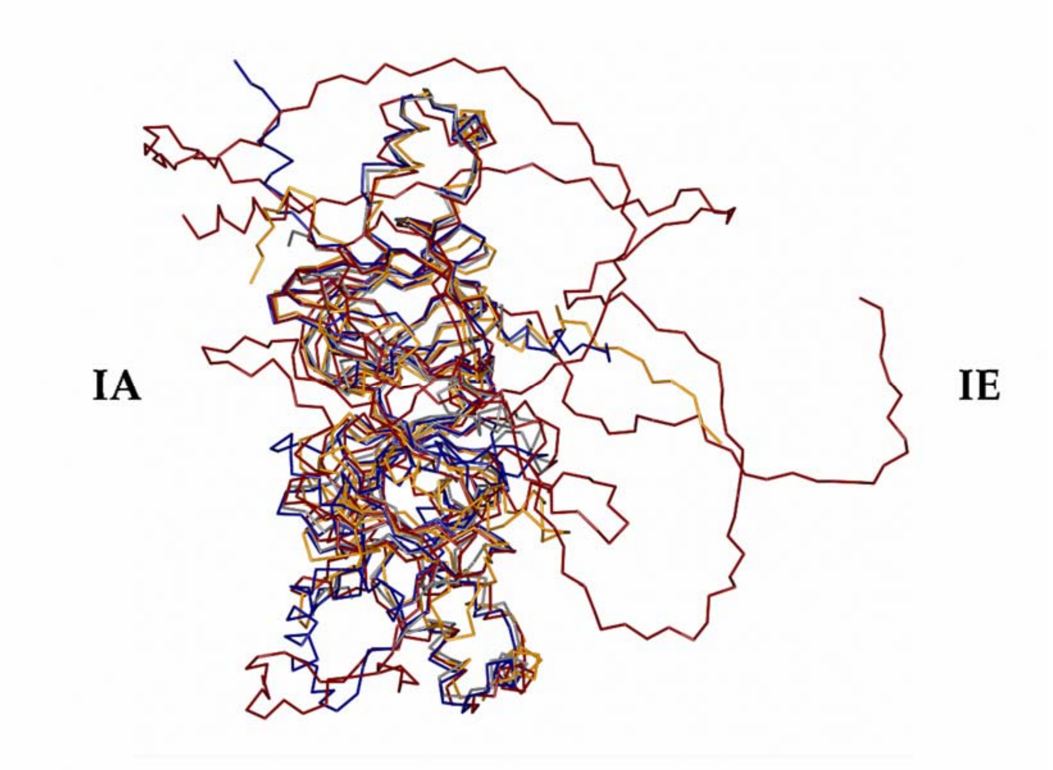

Figure S3. Cα backbone superimposition of Ah11Sn. Gray: Ah11SA (3QAC), blue: Ah11SB, orange: Ah11SC and red: Ah11SHMW. RMSD values in Å: Ah11SB = 1.2, Ah11SC = 1.9, Ah11SHMW = 1.7.

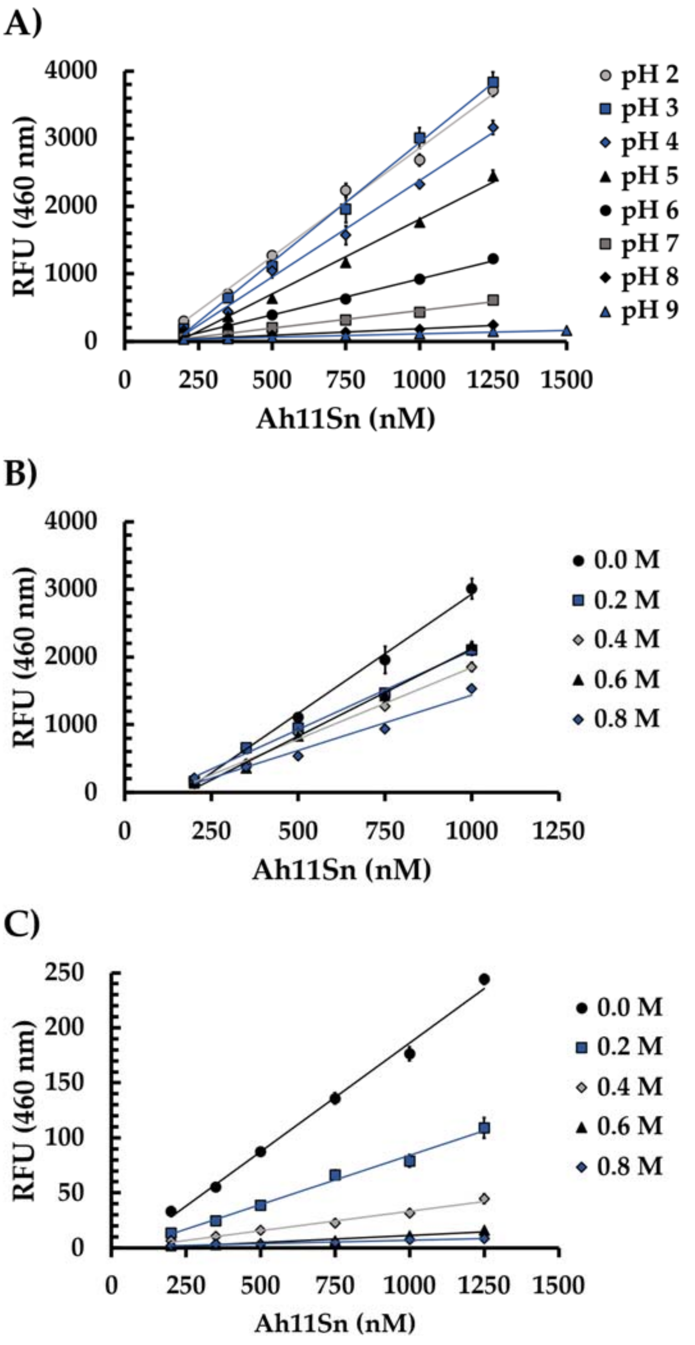

Figure S4. Linear curves from the relative fluorescence units (RFU) versus protein concentrations bounded to ANS. As a function of pH (A). At pH 3 with different NaCl concentrations (B). At pH 8 with different NaCl concentrations (B).

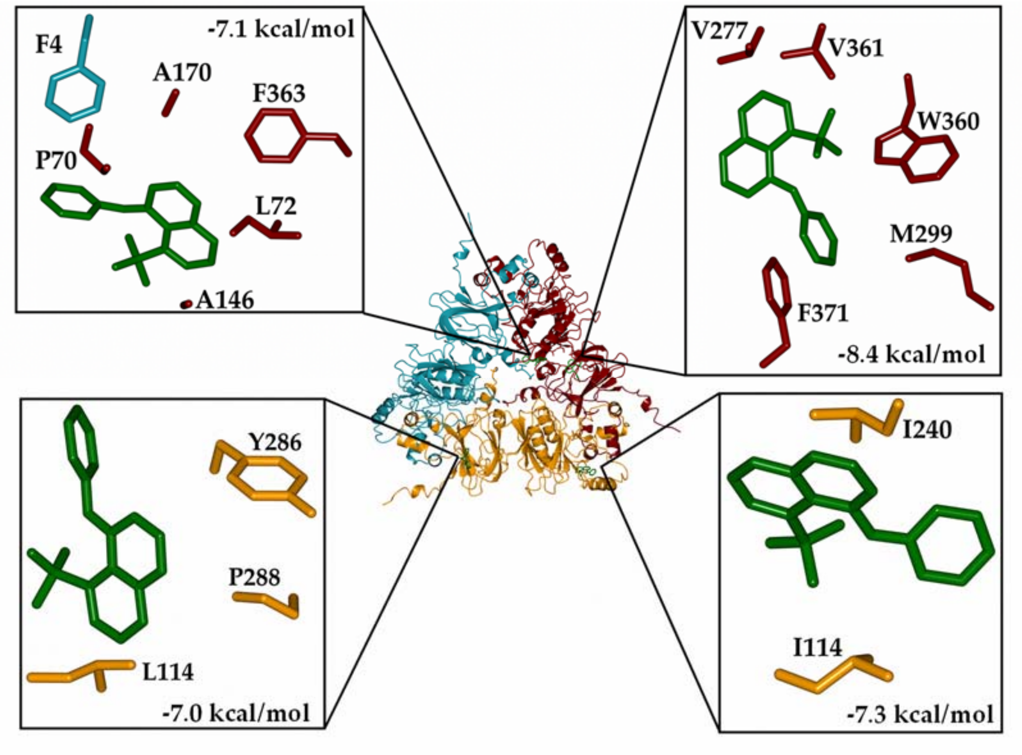

Figure S5. Ah11SB molecular docking for visualization of binding zones of ANS. The ANS molecules are shown in green with their respective energy of interaction. Side chains of each monomer are shown in a different color.

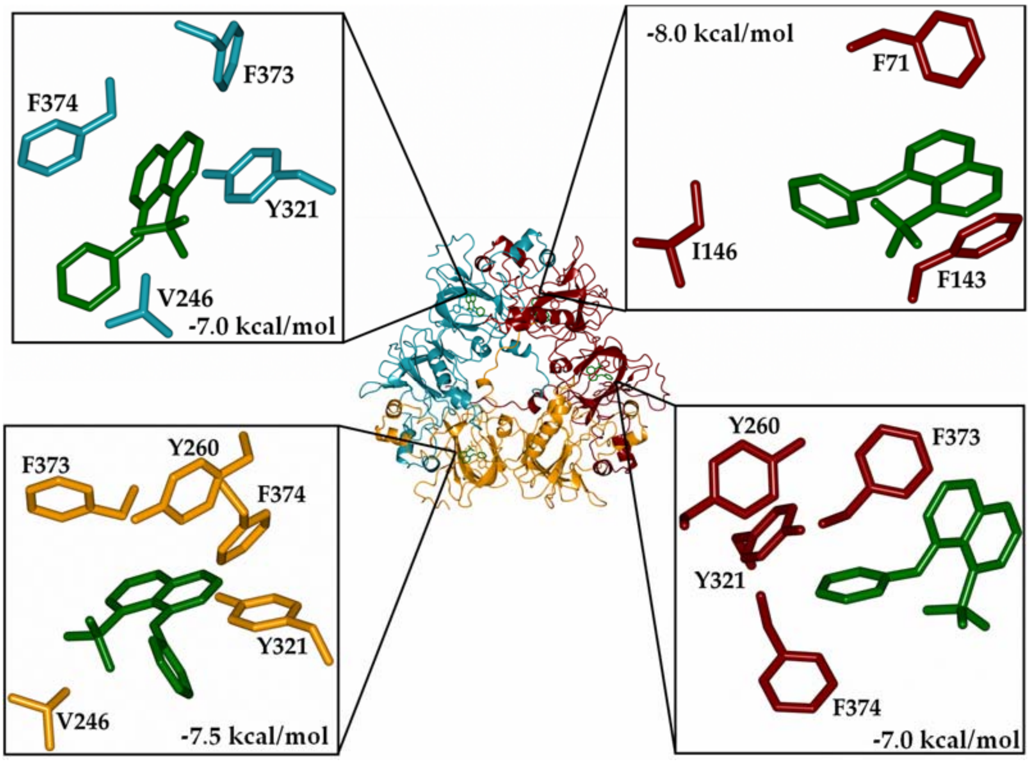

Figure S6. Ah11SC molecular docking for visualization of binding zones of ANS. The ANS molecules are shown in green with their respective energy of interaction. Side chains of each monomer are shown in a different color.

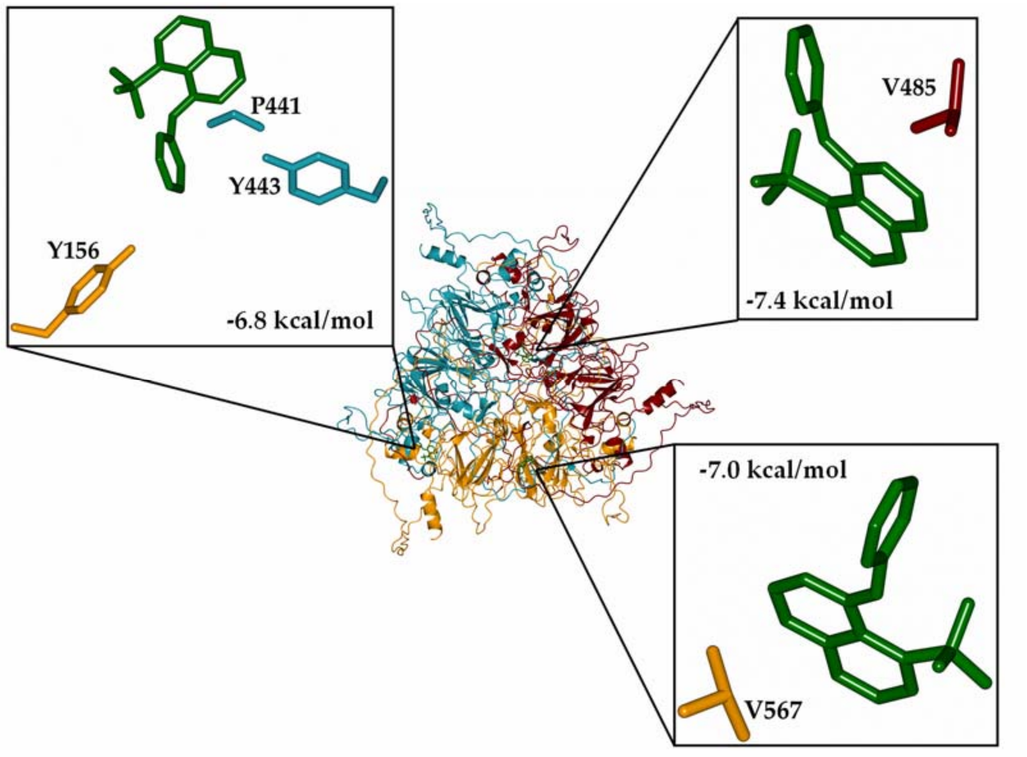

Figure S7. Ah11SAHMW molecular docking for visualization of binding zones of ANS. The ANS molecules are shown in green with their respective energy of interaction. Side chains of each monomer are shown in a different color.

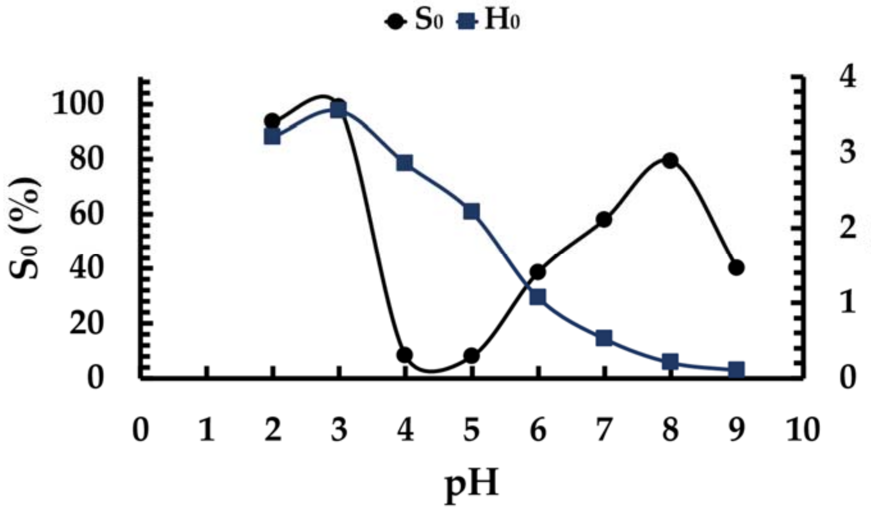

Figure S8. Superposition of surface hydrophobicity and solubility of Ah11Sn at different pHs.
